# Altered postural sway in patients suffering from non-specific neck pain and whiplash associated disorder - A systematic review of the literature

**DOI:** 10.1186/2045-709X-19-13

**Published:** 2011-05-24

**Authors:** Alexander Ruhe, René Fejer, Bruce Walker

**Affiliations:** 1 Murdoch University. Praxis fuer Chiropraktik Wolfsburg, Porschestrasse 1, 38440 Wolfsburg, Germany; 2 Research Department, Spine Centre of Southern Denmark, Hospital Lillebaelt and University of Southern Denmark, Middelfart, Denmark; 3 School of Chiropractic and Sports Science, Murdoch University, Murdoch, 6150, Western Australia, Australia

**Keywords:** Balance, center of pressure, force-plate, neck pain, whiplash, systematic review

## Abstract

**Study design:**

Systematic literature review.

**Objectives:**

To assess differences in center of pressure (COP) measures in patients suffering from non-specific neck pain (NSNP) or whiplash-associated disorder (WAD) compared to healthy controls and any relationship between changes in postural sway and the presence of pain, its intensity, previous pain duration and the perceived level of disability.

**Summary of Background data:**

Over the past 20 years, the center of pressure (COP) has been commonly used as an index of postural stability in standing. While several studies investigated COP excursions in neck pain and WAD patients and compared these to healthy individuals, no comprehensive analysis of the reported differences in postural sway pattern exists.

**Search methods:**

Six online databases were systematically searched followed by a manual search of the retrieved papers.

**Selection Criteria:**

Papers comparing COP measures derived from bipedal static task conditions on a force plate of people with NSNP and WAD to those of healthy controls.

**Data collection and analysis:**

Two reviewers independently screened titles and abstracts for relevance. Screening for final inclusion, data extraction and quality assessment were carried out with a third reviewer to reconcile differences.

**Results:**

Ten papers met the inclusion criteria. Heterogeneity in study designs prevented pooling of the data and no direct comparison of data across the studies was possible. Instead, a qualitative data analysis was conducted. There was broad consensus that patients with either type of neck pain have increased COP excursions compared to healthy individuals, a difference that was more pronounced in people with WAD. An increased sway in antero-posterior direction was observed in both groups.

**Conclusions:**

Patients with neck pain (due to either NSNP or WAD) exhibit greater postural instability than healthy controls, signified by greater COP excursions irrespective of the COP parameter chosen. Further, the decreased postural stability in people with neck pain appears to be associated with the presence of pain and correlates with the extent of proprioceptive impairment, but appears unrelated to pain duration.

## Background

### Rationale

Ideally, the body should be able to generate quick center of pressure (COP) transitions that just exceed the current position of the center of mass (COM) [1] and accelerate it into the opposite direction in order to maintain balance. Any condition effecting the afferent sensory pathways may interfere with this process. The neck is particularly prone to this due to the abundant cervical sensory receptors in joints and muscles [2,3] as well as their central and reflex connections to visual, vestibular and postural control systems [4].

The debate continues however, as to whether the cause of abnormal cervical afferent input is primarily proprioceptive or nocioceptive in nature. Deterioration of this proprioceptive information from the neck may be the determining factor in reducing the accuracy in the sensory integration process. The resulting imprecise estimation of the COM position may then lead to an increase in the safety margin of the adaptive COP shifts with regard to the predicted COM oscillations [5].

The excitation of chemosensitive nociceptors in cervical facet joints and muscles may alter the sensitivity of the muscle spindles by reflex activation of fusimotor neurones [6], leading to a decreased proprioceptive acuity. This effect may be triggered by marked activation of mechano-sensitive nociceptors as occurs in whiplash injuries [7]. Acute "pain inhibition" [8] may be another mechanism where discharge from high-threshold nociceptive afferents interferes with spinal motor-pathways as well as the motor cortex. Pain may also cause an increased pre-synaptic inhibition of muscle afferents [9] as well as affect the central modulation of proprioceptive spindles of muscles [10], causing prolonged latencies. Such alterations may lead to decreased muscle control and result in increased postural sway.

In the case of whiplash associated disorder (WAD), facet joint components may be at risk of injury due to compression during rear-impact accelerations while capsular ligaments are at risk of injury at higher accelerations [11]. Depending on the magnitude of trauma, the resulting impairment of the sensory system is therefore likely to be more pronounced compared to cases of non-specific neck pain (NSNP).

Several attempts have been made to investigate differences in COP sway pattern between people with NSNP and healthy controls by means of forceplate tilting [12], body leaning [13] or vibratory stimulation to structures of the neck [14]. Although these approaches contribute important knowledge to the field, an experimental setup without additional equipment for stimulation or external perturbation that can be applied comprehensively for a broad spectrum of complaints may be of additional use. We previously described that such a simple static setup is not only highly discriminative for non-specific low back pain [15] but also allowed the observation of a linear relationship between the perceived pain intensity and COP sway velocity [16]. If people with NSNP can also be identified by COP measurements during such basic postural tasks, similar relationships are likely and may allow for comparison of postural sway between painful regions.

This literature review will attempt to identify possible differences in COP pattern between people with WAD, people with NSNP and healthy controls that may relate to the mechanisms described above. As COP measures are commonly used in a clinical setting, this will allow the researcher or clinician to put their results into context. To our knowledge no comprehensive systematic review has been conducted to investigate the possible impact of neck pain on COP pattern during bipedal static tasks and the possible association of this effect with pain intensity or disability.

### Objective

The objective of this systematic literature review is to 1) determine if there are significant differences in postural sway between people with NSNP and WAD patients and healthy controls, 2) investigate whether the magnitude of these COP excursions are related to the level of pain perception, previous pain duration or perceived level of disability.

## Methods

### Search

A comprehensive search strategy was developed by identifying all potentially relevant search terms, categorizing these terms into specific search phases and subsequently combining them by using Boolean terms. This search strategy was applied to six different electronic databases: PubMed, MEDLINE, EMBASE, Web of Science, ScienceDirect and the Cochrane library. The date range of publications searched was from January 1980 to January 2011.

The following key words were used in the search strategy: "neck pain", "cervical pain", "whiplash", "WAD", "center of pressure", "COP", "balance", "posture", "postural stability", postural control". The detailed search strategy is available upon contacting the corresponding author.

The hand search included analyzing references cited in studies selected from the original online search. Citation searches of relevant studies were conducted using the PubMed, MEDLINE and ScienceDirect databases.

### Eligibility criteria

Papers were limited to those published in peer-reviewed journals without language restrictions.

The inclusion criteria were: the study investigated force changes over time (postural sway) exhibited by participants with NSNP or WAD derived from bipedal static task conditions on a forceplate, ideally compared to measures of healthy controls. For the purpose of this review, NSNP was broadly defined as pain in the cervical area of musculoskeletal origin in the absence of any neurological symptomatology or serious pathology such as cancer or infection. Induced neck pain in otherwise healthy participants is considered as non-specific neck pain.

The selection criteria for this review does not concern study type as the focus is comparing COP sway data irrespective of the original research purpose of the study. Further, the quality of the various postural sway measures depends on technical aspects of the experimental setup. Therefore all study designs were considered.

We excluded studies with insufficient documentation of patient demographics or experimental setup where this rendered data extraction impossible. In addition, papers that were anecdotal, speculative or editorial in nature or studies that employed dynamic task conditions such as one-leg hopping, walking or some form of translation of the force platform were excluded.

### Information sources

#### Study selection

For the purpose of this review AR acted as the principal reviewer. A colleague (TB) was involved independently in the process of identifying relevant studies but did not participate in further analysis of the finally included papers. Where discrepancies between AR and TB were not reconciled by discussion, a third reviewer was used for a majority decision.

### Data collection process

To standardize the procedure between the reviewers, the main author developed a detailed data extraction sheet to acquire general information on objectives, design, participant's demographics and outcomes. If any title and abstract did not provide enough information to decide whether or not the inclusion criteria were met, the article was included for the full text selection.

With regard to the research question, data extraction was concerned with four main areas regarding the association between neck pain and postural sway: 1) perceived pain intensity, 2) previous pain duration, 3) reported disability levels and 4) the experimental setup applied.

For the latter, we extracted data on 1) sampling duration, 2) number of trials, 3) sampling and cut-off frequency, 4) foot position, 5) visual condition (eyes open/closed), 6) surface condition (firm/compliant) as well as 7) the COP parameters used. These points were based on recommendations for obtaining reliable COP measures [17].

### Summary measures

The principle summary measure in the included studies was differences in means.

### Synthesis of results

We planned to combine the results of the included studies to conduct inter-study comparisons of means and statistical differences. We also planned to do this for NSNP and WAD separately and combined to investigate differences between the two.

## Results

### Study selection

**Figure 1:**
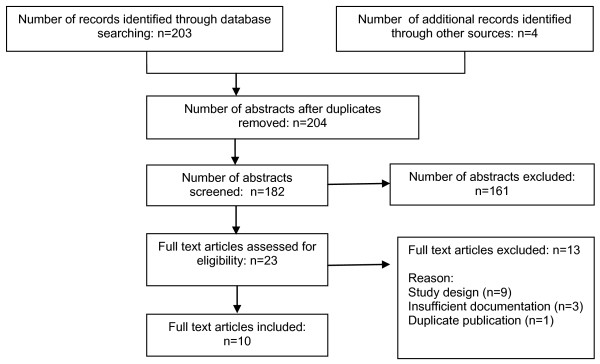
**Flowchart of considered studies**.

Initially, the database search strategy identified 203 studies of which titles and abstracts were screened individually by the reviewers. The application of inclusion/exclusion criteria and consensus by the reviewers on the titles and abstracts eliminated 182 papers. From the titles and abstracts of papers selected (n = 23), full papers were reviewed by the same two reviewers (AR and TB) who applied the inclusion criteria to the full text. Of these, 10 studies met the inclusion criteria and were included in this review (Figure 1). There was full consensus between the reviewers during the selection process of included papers.

### Study characteristics

Combining results was not possible due to the heterogeneous study designs and patient characteristics across the included studies. Therefore only a general trend is noted.

Both subject demographics and health status for all studies are shown in Table 1. The number of symptomatic participants and the matching number of controls was generally small and ranged between seven [18] and fifty [19]. All but two of the included studies (8/10, 80%) enrolled mixed gender groups of healthy and symptomatic participants. The studies employed different age ranges of participants, with 20-40 years being most commonly enrolled (7/10, 70%).

**Table 1:** Participant demographics and health status

Study	Participant status	Gender (n) Female Male		Age in years Mean (SD)	Weight in kg Mean (SD)	Height in cm Mean (SD)
McPartland et al. [18]	NSNP *	6	1	39.1	-	-
	healthy *	4	3	39.4	-	-
Michaelson et al. [21]	chronic NSNP	9	0	40 (9)	73 (18)	165 (7)
	chronic WAD	6	3	44 (10)	79 (14)	171 (10)
	healthy	13	3	41 (9)	70 (14)	168 (8)
Madeleine et al. [23]	chronic WAD *	7	4	33.3 (6.7)	73.4 (11.4)	173.3 (7.2)
	healthy/induced NP	7	4	33.1 (6.8)	68.0 (12.5)	171.5 (6.3)
Treleaven et al. [19]	WAD (dizziness)	38	12	35.6	-	-
	WAD (no dizziness)	38	12	35.8	-	-
	healthy	28	22	29.9	-	-
Storaci et al. [26]	WAD	24	16	28.4 (8.8)	-	-
	healthy	23	17	33.9 (12.7)	-	-
Endo et al. [25]	WAD	19	13	39.0 (10.1)	-	-
	healthy	4	16	37.9 (9.3)	-	-
Treleaven et al. [27]	WAD	15	5	46.5	-	-
	healthy	14	6	49.5	-	-
Field et al. [22]	WAD	24	6	30.3 (1.3)	-	-
	NSNP	23	7	27.9 (1.3)	-	-
	healthy	23	7	26.8 (1.3)	-	-
Poole et al. [24]	NSNP	20	0	65-82	-	-
	healthy	20	0	65-82	-	-
Vuillerme et al. [20]	healthy/induced NP	0	16	22.2 (1.8)	73.0 (11.8)	181.4 (6.4)

NP: neck pain, NSNP: non-specific neck pain, SD: standard deviation, WAD: whiplash-associated disorder

* one patient and one control participants did not participate in COP measurement

- : not described

General shortfalls in the documentation of technical aspects of COP acquirement were apparent. In addition, few authors described the baseline demographics of the participants in appropriate detail, including weight, height, age and gender (3/10, 30%).

There was a marked variation present in the included studies in terms of sampling duration, number of trials or the selection of the COP parameters. The studies often employed a combination of different positional and visual setups in order to investigate postural sway in various challenging positions. The resulting variation in results can be observed irrespective of the COP parameter chosen.

Table 2 shows the study characteristics for sway assessment in people with NSNP. The majority of trials were performed under both eyes open (EO) and eyes closed (EC) condition (4/6, 67%) with only a single repetition (5/6, 83%). Sway area and root mean square (RMS) amplitude were the most commonly used COP parameters.

**Table 2:** Study characteristics and selected COP parameters measured in people with NSNP

Study	Condition	Duration (sec)	Number of trials	Parameter	Neck pain Result (SD)	Healthy controls Result (SD)	p value
McPartland et al. [18]	normal stance EO/EC/F	30	6	absolute	EO/F: 4.2	EO/F: 3.3	p < 0.05
				sway Vel †	EC/F: 4.3	EC/F: 3.4	ns
	narrow stance EO/EC/F	30	6	absolute	EO/F: 4.4	EO/F: 3.7	ns
				sway Vel †	EC/F: 5.3	EC/F: 4.4	ns
Michaelson et al. [21]	narrow stance, EO/EC/F	20	1	sway area (mm^2^)	EO: 105 (73)	EO: 66 (47)	-
					EC: 166 (117)	EC: 109 (65)	-
Madeleine et al. [23]∞	narrow stance, EO/F	45	1	displacement ampl. AP (mm)	EO: ~2.7 *	EO: ~2.1	-
				displacement ampl. ML (mm)	EO: ~1.7 *	EO: ~1.0	-
Field et al. [22]∞	normal stance EO/EC/F/C	30	1	AP RMS amplitude (mm)	EO/F: ~1.3	EO/F: ~1.2	ns
					EC/F: ~1.4	EC/F: ~1.1	p < 0.05
					EO/C: ~2.2	EO/C: ~2.3	ns
					EC/C: ~2.5	EC/C: ~2.4	ns
				ML RMS amplitude (mm)	EO/F: ~2.6	EO/F: ~2.4	ns
					EC/F: ~3.4	EC/F: ~2.8	ns
					EO/C: ~4.1	EO/C: ~4.1	ns
					EC/C: ~6.2	EC/C: ~5.6	ns
	narrow stance, EO/EC/F/C	30	1	AP RMS amplitude (mm)	EO/F: ~3.3	EO/F: ~3.1	ns
					EC/F: ~4.5	EC/F: ~4.0	ns
					EO/C: ~4.5	EO/C: ~4.4	ns
					EC/C: ~7.6	EC/C: ~6.9	ns
				ML RMS amplitude (mm)	EO/F: ~5.2	EO/F: ~5.1	ns
					EC/F: ~6.5	EC/F: ~5.6	p < 0.05
					EO/C: ~6.1	EO/C: ~6.0	ns
					EC/C: ~9.0	EC/C: ~8.2	ns
Poole et al. [24] ∞	normal stance, EC/EO/F/C	30	1	AP RMS amplitude (mm)	EO/F: ~2.3	EO/F: ~3.1	ns
					EC/F: ~5.0	EC/F: ~3.0	p = 0.02
					EO/C: ~5.8	EO/C: ~4.2	p = 0.01
					EC/C: ~7.5	EC/C: ~6.2	ns
		30	1	ML RMS amplitude (mm)	EO/F: ~1.7	EO/F: ~1.8	ns
					EC/F: ~1.9	EC/F: ~1.6	ns
					EO/C: ~3.8	EO/C: ~2.8	ns
					EC/C: ~3.8	EC/C: ~3.5	ns
	narrow stance, EC/EO/F/C	30	1	AP RMS amplitude (mm)	EO/F: ~4.2	EO/F: ~3.6	ns
					EC/F: ~4.4	EC/F: ~4.2	ns
					EO/C: ~5.9	EO/C: ~5.1	p = 0.01
					EC/C: ~8.2	EC/C: ~8.3	ns
		30	1	ML RMS amplitude (mm)	EO/F: ~6.6	EO/F: ~5.0	p = 0.02
					EC/F: ~7.3	EC/F: ~6.0	ns
					EO/C: ~8.3	EO/C: ~7.5	ns
					EC/C: ~10.6	EC/C: ~10.7	ns
Vuillerme et al. [20] ∞	normal stance, EC/F	10	1	Variance (mm^2^)	~19.5 *	~13.5	p < 0.05
				range (mm)	~ 20.3*	~15.5	p < 0.01
				mVel (mm/s)	~17.0 *	~11.3	p < 0.001

∞ The results presented have been extracted from bar-charts.

* Induced neck pain cases and healthy participants are identical.

- : not described

†: unit not described

AP: antero-posterior, BP: bipedal, displ. ampl: displacement amplitude, C: compliant (foam) surface, COP: center of pressure, EC: eyes closed, EO: eyes open, F: firm surface, ML: medial-lateral, mPos: mean position, mVel: mean velocity, ns: not significant (p > 0.05), NSNP: non-specific neck pain, RMS: root mean square, vel: velocity

The study characteristics for trials enrolling WAD patients are presented in Table 3. Only a single recording was used in most cases (6/7, 86%), but in contrast to the NSNP studies, all study designs employed both visual conditions.

**Table 3:** Study characteristics and selected COP parameters measured in people with WAD

Study	Condition	Duration (sec)	Number of trials	Parameter	WAD Result (SD)	Healthy controls Result (SD)	p value
Michaelson et al. [21]	narrow stance, EO/EC/F	20	1	sway area (mm^2^)	EO: 96 (57)	EO: 66 (47)	ns
					EC: 269 (147)	EC: 109 (65)	p < 0.01
Madeleine et al. [23]	narrow stance, EO/EC/F	45	1	displacement ampl. AP (mm)	EO: ~4.6	EO: ~2.1	-
					EC: ~6.0	EC: ~2.5	-
				displacement ampl. ML (mm)	EO: ~2.2	EO: ~1.0	-
					EC: ~3.2	EC: ~1.2	-
Treleaven et al. [19] ∞	normal stance, EO/EC/F/C	30	1	total energy	EO/F: ~0.80	EO/F: ~0.66	ns
					EC/F: ~0.93	EC/F: ~0.70	p < 0.05
					EO/C: ~1.30	EO/C: ~1.15	ns
					EC/C: ~1.52	EC/C: ~1.38	ns
Storaci et al. [26]	unclear stance, EO/EC/F	-	2	sway area (mm^2^)	EO: 136.6 (76.3)	EO: 84.1 (44.8)	-
					EC: 246.3 (127)	EC: 180.1 (102)	-
				path length (mm)	EO: 407.5 (103)	EO: 338 (85.6)	-
					EC: 565.8 (151)	EC: 494.5 (145)	-
Endo et al. [25]	unclear stance, EO/EC/F	60	1	sway area (mm^2^)	EO: 102.8 (109)	EO: 35.0 (14.7)	p < 0.01
					EC: 218.6 (207)	EC: 41.9 (25.2)	p < 0.05
				mVel (mm/s)	EO: 18.6 (12.5)	EO: 13.8 (4.3)	p < 0.001
					EC: 32.8 (22.2)	EC: 17.9 (6.0)	p < 0.001
Treleaven et al. [27] ∞	Normal stance, EO/EC/F/C	-	1	total energy AP	EO/F: ~ 1.2	EO/F: ~0.7	p < 0.01
					EO/C: ~1.6	EO/C: ~1.2	p < 0.01
					EC/F: ~1.4	EC/F: ~0.9	p < 0.01
					EC/C: ~1.9	EC/C: ~1.6	p < 0.01
		-	1	total energy ML	EO/F: ~0.6	EO/F: ~0.2	p < 0.01
					EO/C: ~1.3	EO/C: ~0.7	p < 0.01
					EC/F: ~0.7	EC/F: ~0.2	p < 0.01
					EC/C: ~1.5	EC/C: ~0.9	p < 0.01
	narrow stance, EO/EC/F/C	-	1	total energy AP	EO/F: ~1.2	EO/F: ~1.1	ns
					EO/C: ~1.6	EO/C: ~1.3	p < 0.03
					EC/F: ~1.6	EC/F: ~1.3	p < 0.02
					EC/C: ~1.9	EC/C: ~1.6	p < 0.03
		-	1	total energy ML	EO/F: ~1.5	EO/F: ~1.3	ns
					EO/C:~1.7	EO/C: ~1.6	ns
					EC/F: ~1.7	EC/F: ~1.5	p < 0.02
					EC/C: ~1.9	EC/C: ~1.9	ns
Field et al. [22] ∞	normal stance EO/EC/F/C	30	1	AP RMS amplitude (mm)	EO/F: ~1.4	EO/F: ~1.2	p < 0.05
					EC/F: ~1.5	EC/F: ~1.1	p < 0.05
					EO/C: ~3.1	EO/C: ~2.3	ns
					EC/C: ~3.9	EC/C: ~2.4	p < 0.05
				ML RMS amplitude (mm)	EO/F: ~2.9	EO/F: ~2.4	ns
					EC/F: ~3.5	EC/F: ~2.8	ns
					EO/C: ~5.0	EO/C: ~4.1	p < 0.05
					EC/C: ~7.0	EC/C: ~5.6	p < 0.05
	narrow stance, EO/EC/F/C	30	1	AP RMS amplitude (mm)	EO/F: ~4.2	EO/F: ~3.1	p < 0.05
					EC/F: ~4.8	EC/F: ~4.0	p < 0.05
					EO/C: ~5.3	EO/C: ~4.4	p < 0.05
					EC/C: ~7.9	EC/C: ~6.9	ns
				ML RMS amplitude (mm)	EO/F: ~5.5	EO/F: ~5.1	ns
					EC/F: ~6.3	EC/F: ~5.6	ns
					EO/C: ~6.3	EO/C: ~6.0	ns
					EC/C: ~9.2	EC/C: ~8.2	ns

- : not described

∞ The results presented have been extracted from bar-charts.

ampl: amplitude, AP: antero-posterior, BP: Bipedal, C: compliant surface, COP: center of pressure, EC: eyes closed, EO: eyes open, F: firm surface, ML: medial-lateral, mPos: mean position, mVel: mean velocity, RMS: root mean square, WAD: whiplash associated disorder.

### Reliability of COP data

Table 4 gives an overview of how the studies included meet the ideal experimental setup for reliable data.

**Table 4:** Reliability criteria

Study	Sampling frequency	Cut-off frequency	Duration	Number of repetitions	Visual condition	Surface	Total
Recommended	~100 Hz	10 Hz	≥ 90 sec	3-5	eyes closed	firm	
McPartland et al. [18]	+	0	0	+	+	+	++++
Michaelson et al. [21]	0	0	0	0	+	+	++
Madeleine et al. [23]	+	+	0	0	+	+	++++
Treleaven et al. [19]	0	0	0	0	+	+	++
Storaci et al. [26]	0	0	0	0	+	+	++
Endo et al. [25]	0	0	0	0	+	+	++
Treleaven et al. [27]	0	0	0	0	+	+	++
Field et al. [22]	0	0	0	0	+	+	++
Poole et al. [24]	0	0	0	0	+	+	++
Vuillerme et al. [20]	0	0	0	0	+	+	++

With the exception of one paper that only measured postural sway under visual deprivation [20], all of the studies included assessed COP with both eyes open and eyes closed. No study applied best practice experimental setup throughout.

### Pain assessment

All symptomatic participants experienced pain at the time of recording. About 75% of studies described the total neck pain duration prior to the COP measurements whereby the pain history ranged from acute, induced pain to 97 (SD 68) months. Of these studies, half (5/8, 63%) assessed both the duration and the perceived pain intensity by using either the visual analogue scale (VAS) [19-22] or the 11-box numeric rating scale (NRS-11) [23].

The perceived pain levels varied between the studies (Table 5). The pain intensity of WAD patients ranged between VAS 2.2 (SD 0.9) [22] and 4.9 (SD 2.3) [21], indicating mild to moderate pain. Individuals with NSNP perceived pain within a similar range and rated their intensity from VAS 3.2 (SD 0.4) [22] to 5.2 (SD 1.6) [21].

**Table 5:** Pain definition, intensity and characteristics of included studies

Study	WAD	NSNP	Pain presence in months (SD)	Pain present at time of trial	Pain intensity evaluation (pre-trial)	Score mean (SD)
McPartland et al. [18]		X	-	yes	-	-
Michaelson et al. [21]	X		87 (77)	yes	VAS	4.9 (2.3)
		X	97 (68)	yes	VAS	5.2 (1.6)
Madeleine et al. [23]	X		≥ 6	yes	NRS-11	6.0 (0.7)
		X	induced	yes	NRS-11	2.6-4.5 (0.5)
Treleaven et al. [19]	X		-	yes	VAS	2.8
	X		-	yes	VAS	4.1
Storaci et al. [26]	X		-	-	-	-
Endo et al. [25]	X		6	yes	-	-
Treleaven et al. [27]	X		17	yes	-	-
Field et al. [22]	X		≥ 3	yes	VAS	2.2 (0.9)
		X	≥ 3	yes	VAS	3.2 (0.4)
Poole et al. [24]		X	> 5	yes	VAS	-
Vuillerme et al. [20]		X	induced	yes	VAS	7.1 (1.7)

- : not reported, NDI: Neck Disability Index, NSNP: non-specific neck pain, SD: standard deviation, WAD: whiplash associated disorder

∞ : induced pain

Visual Analogue Scale (VAS) ranging from 0-10: 0-2: light pain, 3-5: light to moderate pain, 6-7: moderate to intense pain, 8-10: unbearable pain.

### Neck pain and postural sway

Generally there was a great variability in the reported COP measures. The results of the included studies indicated that patients with any form of neck pain exhibited a greater postural instability than healthy controls, a difference that was more pronounced in WAD patients.

In people with NSNP, a significant difference compared to healthy individuals was only observed in a minority of recordings (9/38, 24%) across all positional and visual conditions. Statistical significance was reached only in normal stance under visual deprivation on a firm surface [20,22,24] as well as with open eyes on both firm [18] and compliant surface [24]. In narrow stance the differences reached p ≤ 0.05 with eyes open [24] and closed [22] on a firm surface as well as on a foam pad with eyes open [24]. One study failed to report levels of significance [21].

In cases of acutely induced neck pain, a marked immediate increase in postural sway could be observed. While Vuillerme et al. [20] found a significantly increased mean sway velocity and area, no *p*-values were calculated for the study by Madeleine et al. [23] (Table 2).

People with WAD also showed an increased postural sway, indicated by a greater COP sway area [21,25,26], total energy [19,27], root mean square (RMS) amplitude and mean sway velocity [22,25]. In contrast to NSNP patients, the variance in COP excursion compared to healthy controls was significant in the majority of experimental setups, although two studies did not report levels of significance [23,26]. The increase in postural sway in antero-posterior (AP) direction was more significant than in the medio-lateral (ML) plane [22,23] (Table 3).

### Disability assessment

Only three studies [19,22,24] assessed the level of disability in neck pain patients using the neck disability index (NDI) [28]. The NSNP patients scored NDI disability percentages between 21.5% (SD 1.4) [22] and 23.95% (SD 2.3) [24] while people with WAD had higher levels of impairment at 36.9% (SD 2.8) [22]. Scores from 21-40% indicate moderate disability.

## Discussion

### Summary of evidence

The heterogeneous study designs and experimental setups did not allow pooling of data or any direct comparison of results across the studies. In addition, the poor overall documentation of the experimental setups, particularly with regards to participant demographics and technical aspects such as sampling frequency and cut-off frequency, impaired full interpretation. However, despite the great variability there was enough consistency in results to show that patients suffering from NSNP and WAD exhibit an increased COP sway compared to healthy individuals, especially in AP direction. Unfortunately, the magnitude of these differences in postural sway cannot be summarily expressed in terms of specific percentages or values. As a result, only a general trend is noted.

As we outlined in a previous systematic review [17], the reliability of COP measurements is primarily determined by the six main factors (Table 4). Although only two of the included studies fulfilled more than half of the recommended reliability criteria [18,23], it is worth bearing in mind that studies considering less than all six criteria may still present fairly reliable results.

While a general trend towards decreased postural stability can be observed irrespective of the origin of the pain, the altered sway pattern appears to correlate with the associated degree of proprioceptive impairment. This is signified by the generally greater COP excursions in WAD cases [21-23] where damage to proprioceptive structures and neck musculature due to the sustained trauma may be expected. In addition, higher pain intensities or the underlying neurological or vestibular impairments observed in several studies [21,25] may be the determining factor in the reported highly significant differences in sway pattern compared to healthy controls. The lack of comparable data does not allow the interpretation of previous pain duration or associated perceived disability in this context. While some WAD patients may have also been included in NSNP studies, it appears unlikely that this affected the overall results.

We have decided to include studies using induced pain in our review. While this cannot be considered similar to (chronic) NSNP, it may nevertheless mimic many alterations in sensorimotor functions documented in acute clinical pain conditions, although it should be noted that it does not replicate any potential long term neurological adaptation. Both experiments resulted in significantly altered sway pattern which may underline the role of acute "pain inhibition" [8] in the observed postural response. However, the COP sway area measured was nevertheless smaller than reported in people with WAD [23] which may underline the likely role of proprioceptive impairment associated with the pain in the development of COP excursions of larger magnitude.

Visual deprivation caused an increase in postural sway in numerous studies of healthy participants [29-32] and has shown to be a major challenge to the balance systems in studies investigating the effect of non-specific low back pain on postural stability [29,33,34]. Nevertheless, statistically significant differences were not found in a number of NSNP studies (Table 2). In addition to issues arising from the experimental setups and the generally small sample sizes of seven [18] to thirty [22] symptomatic participants, the variations in the perceived pain intensities may offer an explanation.

Pain severity has shown to be a determining factor in non-specific low back pain cases [16] where a significant, linear increase in postural sway was observed beginning at a NRS-11 score of 5. If this can be applied to NSNP patients as well, low pain intensities at the time of recording such as those reported by Field et al. [22] may well explain the fact that no significant differences could be identified, while patients suffering from more severe pain exhibited significantly increased postural sway compared to healthy controls [20].

If rather small differences in COP measures between the groups can be anticipated, the choice of appropriate sway parameters is important. However, only Vuillerme et al. [20] and Endo et al. [25] used mean velocity (mVel), a parameter that has shown both consistently high reliability [17] and discriminative value in pain conditions [15]. Despite a small sample size and low scores for the reliability of the experimental setup, they found highly significant differences with eyes open [25] and under both visual conditions [20].

The effect of ageing can be observed when comparing the studies by Field et al. [22] and Poole et al. [24]. Although the methodologies are very similar, varying results were reported. This may be explained by the fact that the latter enrolled elderly patients (65-82 years compared to 27-30 years). Older individuals exhibit increased COP excursions [35] and any pre-existing deficits in proprioception associated with ageing may add to the alterations caused by the neck pain.

Overall, the lack of data available, no conclusions can be drawn regarding a possible relationship between postural stability and perceived pain or disability levels. For the same reason, no conclusion about the effect of impairments in cervical ROM is possible.

### Clinical considerations

At this point, there are several important limitations to the application of COP measures in the assessment of postural sway in a clinical setting:

Although the results tempt us to hypothesize a correlation between the magnitude of COP excursions and the extent of damage to proprioceptive structures, the causative factor for the altered postural sway pattern remains largely unclear in people with WAD and NSNP. The question still remains whether the increased COP excursions are predominantly related to the previously described physiological changes due to chronic pain perception, acute or chronic damage to proprioceptive structures in the neck or acute "pain inhibition" [8]. If the latter mechanism is mainly responsible or if the proprioceptive impairment is of acute and reversible nature, monitoring neck pain patients during their treatment and rehabilitation process may aid as an objective tool in assessing the patient's progress. If long-term neuro-physiological changes are primarily involved, individually varying recovery time frames may render such measurements less useful.

Finally, the data available is insufficient to determine whether some form of correlation between the neck pain intensity, its duration or the perceived disability and the magnitude of postural sway exists. As a linear relationship between pain intensity and COP sway velocity has been demonstrated in patients with non-specific low back pain [16], further research is necessary to investigate whether this also applies to people with neck pain. If this can be established COP may have a clinical role as an instrument of measurement for neck pain patients.

### Limitations

Although employing two reviewers to individually search the literature constitutes a major strength of this review, there are limitations. For example, the search strategy was limited to six key databases which might not have identified all relevant papers. To overcome this, a dynamic search strategy was employed with selected hand searches of reference lists. Due to the aim of this review, only COP measures derived from bipedal static tasks were included.

## Conclusions

Patients with neck pain of both whiplash associated disorder and non specific neck pain exhibit greater postural instability than healthy controls. This difference is more pronounced under visual obstruction and may be attributed to either acute pain inhibition or diminished proprioceptive input from the cervical spine and neck muscles due to long-term neurological adaptations although additional cognitive and behavioral factors cannot be ruled out. People with WAD show greater COP excursions than NSNP patients and this may be due to the potentially increased damage to cervical proprioceptive structures associated with the sustained neck trauma,

While the presence of pain itself appears associated with increased postural sway, there is insufficient data to suggest a relationship between pain intensity, previous pain duration or the level of perceived disability and the magnitude of COP excursions.

## Competing interests

The authors declare that they have no competing interests.

## Authors' contributions

AR and Tino Bos (TB) carried out the literature search and both participated in the selection of the included papers. AR drafted the manuscript and performed the statistical analysis. RF and BW helped with the design of the study and drafting the manuscript. All authors read and approved the final manuscript.
